# Matrix-based vector representations in neural networks for classifying molecular biology data

**DOI:** 10.1093/bioadv/vbaf251

**Published:** 2025-11-08

**Authors:** Loris Nanni, Sheryl Brahnam, Daniel Fusaro

**Affiliations:** Department of Information Engineering, University of Padova, Padova 35131, Italy; Department of Information Technology and Cybersecurity, Missouri State University, Springfield, MO 65897, United States; Department of Information Engineering, University of Padova, Padova 35131, Italy

## Abstract

**Summary:**

Selecting an appropriate classifier is essential for achieving accurate classification. In this study, we propose novel neural network (NNs)-based alternatives to standard classifiers as support vector machines. NNs, particularly convolutional neural networks and transformer networks, have shown exceptional performance in processing image data. To leverage this capability, we explore methods for transforming 1D vector data into 2D matrix representations, enabling the application of NNs pre-trained on large-scale image datasets. Specifically, we introduce a new data restructuring technique based on Wigner transforms, and we compare many methods proposed in the literature. The effectiveness and robustness of our approach are assessed using various benchmark datasets, from peptide classification to DNA barcoding classification, demonstrating consistently strong performance.

**Availability and implementation:**

All source code and related resources used in this work are made publicly available at https://github.com/LorisNanni/Matrix-Representation-of-Vectors-in-Neural-Networks-for-Data-Classification.

## 1 Introduction

Support vector machines (SVMs) remain one of the leading solutions in many domains due to their user-friendliness, strong generalization capabilities, and robust performance. Driven by the growing interest in harnessing the high predictive performance of deep learning models, there has been a surge of research aimed at transforming feature vectors traditionally used for training SVMs, or other standard machine learning methods, into visual representations, such as 2D or 3D images (Sharma *et al.* 2023). Recent advancements, such as the DeepInsight method (Sharma *et al.* 2019), have demonstrated the feasibility of converting non-image data into organized image formats, thereby enabling convolutional neural networks (CNNs) to classify diverse types of inputs, including feature sets. DeepInsight has even evolved to incorporate transformer models (Gokhale *et al.* 2023). Many approaches are detailed and compared in Sharma *et al.* (2023) and Zhu *et al.* (2021). Similar concepts have been applied in many domains; i.e. researchers have mapped molecular descriptors and fingerprint features into 2D feature maps for CNN processing (Shen *et al.* 2021), treated molecular data as 2D images to leverage image recognition networks (Yoshimori 2021).

The principal contributions of our research are summarized as follows:

1. We perform a comprehensive analysis of various methods for integrating 2D feature vector representations with pre-trained nets.
2. We introduce an ensemble classifier that significantly outperforms the standalone SVM.
3. We propose a novel technique for representing a feature vector as an image based on the Wigner transform.
4. All source code and related resources used in this work are made publicly available at https://github.com/LorisNanni/Matrix-Representation-of-Vectors-in-Neural-Networks-for-Data-Classification

## 2 Materials and methods

For each method, we ensemble fifteen neural networks (NNs) by averaging their outputs. Prior to every training run, we randomly permute the input feature dimensions and then apply a given three-channel data-conversion transform. We have used the following approaches for representing the vector as a 3-channel matrix for feeding a neural net:

1. Reshape, as proposed in Nanni *et al.* (2023);
2. DeepIns, deep insight as proposed in Sharma *et al.* (2019);
3. IGTD, Image Generator for Tabular Data as proposed in Zhu *et al.* (2021);
4. CWT, continuous wavelet as proposed in Nanni *et al.* (2025b);
5. DWT, discrete wavelet as proposed in Nanni *et al.* (2025a);
6. Wigner, as proposed here.

In our investigation, we have tested these approaches using ResNet50 and MobileNetV2, both pre-trained on ImageNet. ResNet50 is trained using stochastic gradient descent with momentum, with features normalized between 0 and 255 before applying the vector-to-3-channel methods. In contrast, MobileNetV2 is trained with Adam and does not use this normalization step in order to encourage diversity with respect to ResNet50. Both networks use a learning rate of 0.001 and a batch size of 30. ResNet50 is combined with a stochastic method for selecting the activation function for each layer, as described in Nanni *et al.* (2023), while MobileNetV2 consistently uses ReLU. The networks are trained for 30 epochs; however, due to computational constraints, we evaluate performance on the “Unseen” dataset using 10 epochs. We select the trained net that achieved the highest performance on the training set, rather than the one obtained at the final epoch.

SVM were implemented using the widely recognized libSVM library (https://www.csie.ntu.edu.tw/∼cjlin/libsvm/), hyperparameter selection was carried out via grid search on the training set, incorporating a 5-fold cross-validation procedure internal to the training data. During the training and testing of the SVMs, feature values are normalized to the [0,1] range using only the training data for finding the normalization parameters. Notably, we observed that many studies introducing novel classification approaches often fail to adequately optimize SVM, frequently relying on default parameters.

### 2.1 Wigner–Ville distribution

The Wigner–Ville Distribution (WVD) of a single mean-zero time series *x* is given by first expressing the autocorrelation function


(1) $$R_{xx}(t_{1},t_{2})=E\left[x\left(t_{1}\right)x^{*}\left(t_{2}\right)\right],$$

where $x^{*}$ is the complex conjugate of *x*, in terms of the average time $t=\frac{t_{1}+t_{2}}{2}$ and time lag $\tau =t_{1}-t_{2}$, to obtain


(2) $$R_{xx}(t_{1},t_{2})=E\left[x\left(t+\frac{\tau }{2}\right)x^{*}\left(t-\frac{\tau }{2}\right)\right]$$

and then Fourier transforming the lag τ.

The WVD can be defined, in terms of *t* and frequency *f*,for a stochastic process *x*, as


(3) $$\text{WVD}(t,f)=\int_{-\infty }^{+\infty }R_{xx}\left(t,\tau \right)e^{-j2\pi ft}d\tau .$$

Unlike other time-frequency analysis techniques, such as the short-time Fourier transform (STFT), WVD does not suffer from spectral leakage: it does not rely on a window function, allowing for a more accurate representation of the signal’s true frequency content.

As a downside, in presence of signals with several frequency components the WVD suffers from the so-called cross terms (Pachori and Nishad 2016). For this reason, the original formulation of Equation (3) is rarely used in practice. However, by adding two carefully chosen and tuned window functions we are able to suppress the cross terms while keeping a high resolution of time and frequency. The main technique adopting this strategy is a variant of WVD, called smoothed WVD (SWVD). In our work, we use the point-wise absolute value of the SWVD defined for a discrete signal *x* of *N* samples, the SWVD becomes


(4) $${\text{SWVD}}^{g,H}(n,f)=\sum_{m=-N}^{N}g(n)H(f)R_{xx}(n,m)e^{-j2\pi fm}.$$

where *g* and *H* are independent windows to smooth in time and frequency, respectively.

Computing the SWVD of the signal eliminates the cross terms between the positive and negative regions of the spectrum. Given the time-frequency analysis technique presented, we propose an ensemble of networks. The ensemble is trained on 3-channel matrix obtained from 1D feature vectors, i.e. signals, by applying a WVD transformation using a Kaiser window function with random increasing size. Given a signal x(n), the Wigner–Ville tensor $X_{\text{Wigner-Ville}}\in R^{N\times N\times 3}$ is computed as follows:


(5) $$X_{\text{Wigner-Ville}}(:,:,i)={\text{SWVD}}_{M_{i}},$$

for i∈{1,2,3}, where ${\text{SWVD}}_{M_{i}}$ is computed from Equation (4) using a Kaiser window of size $M_{i}$ for both time and frequency windows. Each $M_{i}$ is sampled as follows:


(6) $$\begin{array}{c} M_{1}∼U(0.3N,0.5N),\\ M_{2}∼U(0.5N,0.7N),\\ M_{3}∼U(0.7N,0.9N). \end{array}$$

Clearly, $M_{1}\leq M_{2}\leq M_{3}$. We chose increasing window sizes in order to break the symmetry between two networks with swapped window size in order to enhance the network diversity.

In the DNA barcoding datasets, where a long feature vector describes each pattern, the feature vector is partitioned into four equilength segments, and the aforementioned method is applied independently to each segment. The resulting four output matrices are subsequently combined to form a single matrix, where each output matrix constitutes one quadrant of the final composite matrix.

Compared with standard spectrograms, the WVD’s quadratic nature yields sharper localization of instantaneous frequencies and energies. You obtain a 2D matrix in which transient or multi–component features appear as well-defined ridges rather than blurred blobs. The WVD’s superior concentration makes subtle time–frequency patterns more distinguishable to downstream wavelet filters, a benefit especially evident in signals with rapidly changing spectral content (Torrésani 1999). While DWT excels at multi‐resolution analysis for capturing both coarse and fine details using a given kernel, it may miss joint time–frequency signatures. By constructing a WVD matrix, you convert the signal into a domain where both temporal and spectral features co‐exist in a single representation (Boashash 2015).

## 3 Experimental results

We use the following datasets:

1. Bioactive peptides (Zhang *et al.* 2024): Anticancer peptides (ACPs), particularly, have become a prominent focus of research due to their ability to induce tumor cell apoptosis and suppress both tumor growth and metastasis. Another major area of interest in bioactive peptide research is Angiotensin-Converting Enzyme (ACE) inhibitory peptides; they can effectively lower blood pressure and improve cardiovascular health. We use the ACP and ACE datasets with the same split training/test tested in Zhang *et al.* (2024). Each peptide is described using the ESM-2 net, 320 features for each peptide, as detailed in Zhang *et al.* (2024). The ACP dataset comprises 1722 peptides (861 positives and 861 negatives), and ACE is composed of 1020 peptides (394 positives and 626 negatives).
2. Aptamer dataset (APT) (Shin *et al.* 2023): aptamers are biomolecules composed of single-stranded DNA or RNA that fold into complex tertiary structures. This dataset aims to find aptamers that bind specifically to target proteins. We use the same dataset and training/test sets proposed in Shin *et al.* (2023): the training set has 580 positive and 1740 negative RNA aptamer-protein pairs, and the test set has 145 positives and 435 negatives. Each pattern is described by 290 features shared by the authors of Shin *et al.* (2023).
3. DNA barcoding datasets: Two datasets were used in this study. These include the Beetle and Fish datasets, both originally introduced in Yang *et al.* (2022). The Beetle dataset contains 615 sequences from 123 beetle species. The Fish dataset has 1070 sequences representing 214 fish species. Each DNA barcoding sequence is described by features proposed in Nanni *et al.* (2025a) (3250 features). We used a 10-fold cross validation as testing protocol.
4. Unseen: In Arias *et al.* (2025), a dataset comprising 67 267 barcodes from 1653 arthropod species spanning 500 different genera is proposed. Each species is represented by a minimum of 20 and a maximum of 50 barcodes. The dataset is divided into training, testing, and validation subsets. Additionally, to provide a more challenging evaluation, an unseen partition is introduced. This partition contains 4278 barcodes from 1826 arthropod species that do not appear in any other subset. The goal for this unseen dataset is genus-level classification. Each DNA barcoding sequence is described by features obtained using Safari *et al.* (2025).

As performance indicator, we have used area under the ROC curve (AUC) for the 2-class problems (ACE, ACP and APT) and the accuracy for the DNA barcoding multi-class datasets. In Tables 1 and 2 we report the performance of the different approaches for building the 3-channel matrices for ResNet50/MobileNetV2. Moreover, we report:

**Table 1.** Comparison of AUC scores across ACE, ACP, and APT datasets, bold values are the best performance.^a^

Method	ACE		ACP		APT	
	ResNet50	MobileAdam	ResNet50	MobileAdam	ResNet50	MobileAdam
Reshape	91.85	93.70	82.94	82.94	82.28	80.75
DeepIns	94.69	95.25	83.06	81.12	78.86	†
IGTD	91.92	94.28	82.30	82.74	83.29	81.31
CWT	94.55	95.24	83.25	83.16	78.16	78.08
DWT	94.39	95.05	82.97	83.02	82.08	77.66
Wigner	91.76	93.97	81.71	82.40	83.00	80.03
Fusion_old	91.92	94.20	82.81	83.06	82.84	81.43
Fusion	93.44	95.18	83.16	83.35	82.16	80.73
SVM	96.24		81.16		83.70	
RF	95.10		80.82		82.10	
AdaBoost	95.56		81.56		83.50	
MLP	93.54		80.10		80.56	
CNNe	94.56		**83.38**		82.14	
SVM+CNNe	95.88		82.62		83.70	
2×SVM+CNNe	**96.25**		82.22		**83.92**	
Zhang *et al.* (2024)	96.22		82.56			

a The symbol † indicates no convergence of the method.

**Table 2.** Accuracy in the DNA barcoding datasets, bold values are the best performance, the results related to the unseen datasets are from Safari *et al.* (2025).

DNA barcoding	Fish	Beetle	Unseen
DWT	95.7	95.9	80.5
Wigner	94.8	97.0	81.3
Fusion	96.1	97.2	82.9
SVM	96.0	97.6	88.5
RF	96.2	96.1	86.5
AdaBoost	95.3	96.2	85.9
MLP	94.1	95.0	84.6
SVM+CNN	96.3	97.8	86.7
2×SVM+CNN	96.4	98.0	**88.9**
Yang *et al.* (2022)	96.3	98.1	
eDNA (Nanni *et al.* 2024)	**96.7**	98.2	
4×eDNA + SVM + CNN	**96.7**	**98.4**	
4×eDNA + 2×SVM + CNN	**96.7**	**98.4**	
BLAST			83.9
DNABERT (*k* = 6)			48.1
DNABERT-2			23.5
DNABERT-S			30.6
HyenaDNA-tiny (d256)			37.5
Nucleotide Transformer			40.1
BarcodeBERT			78.5
BarcodeMAE			88.5

1. Fusion_old, it is the method proposed in Nanni *et al.* (2023);
2. Fusion, the fusion by mean rule of the NNs trained using the six methods (Reshape, DeepIns, IGTD, CWT, DWT and Wigner) for feeding ResNet50/MobileNetV2;
3. SVM, where the LibSVM tool is used;
4. RF, Random Forest, ensemble of 100 trees, as for the SVM, hyperparameters are optimized through grid search on the training set.
5. AdaBoost, hyperparameters (e.g. max depth = 1 and number of estimators) are optimized through grid search on the training set;
6. MLP, 1d input network, hyperparameters (e.g. number of fully connnected layers and number of hidden neurons) are optimized through grid search on the training set;
7. CNNe, the mean rule between the ResNet50 fusion and the MobileNetV2 fusion;
8. w_1_X+w_2_Y, weighted mean rule between X with weight w_1_ and Y with weight w_2_, if w_1_ or w_2_ are not specified its value is 1. Before the fusion the score of X and X are normalized to mean = 0 and standard deviation = 1.

In order to highlight the proposed methods in the tables, we have shaded the corresponding rows in gray. Since the proposed method depends on the dataset being used (and consequently on the available features), multiple rows appear in gray: each of them corresponds to the proposed approach applied to a different dataset. To reduce computational time for the DNA barcoding test, we used only the MobileNetV2 network and a subset of the methods for generating convolutional network inputs. For these datasets, the method named “Fusion” is the mean rule among the networks trained using DWT and Wigner.

There is no single clear winner among the different input encoding methods or between ResNet and MobileNet architectures. Performance varies across datasets, suggesting that the optimal choice is context-dependent. However, fusion strategies consistently offer the best trade-off, as they balance strengths across methods. As highlighted in previous works, it is crucial to assign greater weight to SVMs when used in combination with these approaches. The use of weighted fusion yields equal or superior results to stand-alone SVMs across all tested datasets, an outcome that is far from negligible even if the improvement is not high. To minimize any risk of overfitting, all methods for vector to 3-channels were tested using an identical set of hyperparameters across datasets.

For the bioactive peptide datasets, we reused the original code provided by the authors of Zhang *et al.* (2024) and retrained under our experimental setup. In the original paper, the authors proposed using ensembles with different weighting schemes for the two datasets. In contrast, we used a single ensemble configuration, the one that achieved the best trade-off across both datasets, to align more closely with our evaluation protocol, which consistently applies the same ensemble without any dataset-specific adaptations. It is important to note that performance in Yang *et al.* (2022) is achieved by combining DNA and image data, making direct comparison unfair. Nevertheless, our DNA-only approach still outperforms (Yang *et al.* 2022), which relies on both modalities. The various methods reported for the Unseen dataset are from Safari *et al.* (2025); many recent foundation models have been evaluated on this dataset, and even in this dataset, our ensemble surpasses the current state of the art. It is important to note that, in these problems, the upper bound of achievable performance will not correspond to an AUC of 1 or an accuracy of 100%, as the available information in the patterns does not permit such results. Therefore, any improvement in performance, whether relative to a fine-tuned LibSVM, should be considered attractive.

## 4 Conclusions

In this study, we investigated the training of NNs in bioinformatic/computational biology applications using input matrices obtained by reshaping original feature vectors. We compared and combined NN ensembles with SVMs across these datasets and found that our method yielded equal or superior results. We hope that the findings presented here will inspire further research into adapting conventional NN architectures for the classification of feature vector data in the field of bioinformatics. In future work, we plan to evaluate additional datasets and explore a wider range of NN architectures, including the application of graph NNs to molecular structures.

## References

[vbaf251-B1] Arias PM , Sadjadi N, Safari M et al Barcodebert: transformers for biodiversity analysis. https://arxiv.org/abs/2311.02401. 2025. 10.1093/bioadv/vbag054 PMC13008329 41878470

[vbaf251-B2] Boashash B. Time-Frequency Signal Analysis and Processing: A Comprehensive Reference. Amsterdam, Netherlands: Academic Press, 2015.

[vbaf251-B3] Gokhale M , Mohanty SK, Ojha A. Genevit: gene vision transformer with improved deepinsight for cancer classification. Comput Biol Med 2023;155:106643. 36803792 10.1016/j.compbiomed.2023.106643

[vbaf251-B4] Nanni L , Brahnam S, Loreggia A et al Heterogeneous ensemble for medical data classification. Analytics 2023;2:676–93.

[vbaf251-B5] Nanni L , Cuza D, Brahnam S. Ai-powered biodiversity assessment: species classification via dna barcoding and deep learning. Technologies (Basel) 2024;12:240.

[vbaf251-B6] Nanni L , Gobbi MD, Junior RDAM et al Advancing taxonomy with machine learning: a hybrid ensemble for species and genus classification. Algorithms 2025a;18:105.

[vbaf251-B7] Nanni L , Maritan N, Fusaro D et al Insect identification by combining different neural networks. Expert Syst Appl 2025b;273:126935.

[vbaf251-B8] Pachori RB , Nishad A. Cross-terms reduction in the Wigner–Ville distribution using tunable-q wavelet transform. Signal Processing 2016;120:288–304.

[vbaf251-B9] Safari M , Arias PM, Lowe SC et al Enhancing DNA foundation models to address masking inefficiencies. https://arxiv.org/abs/2502.18405. 2025.

[vbaf251-B10] Sharma A , Lysenko A, Boroevich KA et al Deepinsight-3d architecture for anti-cancer drug response prediction with deep-learning on multi-omics. Sci Rep 2023;13:2483. 36774402 10.1038/s41598-023-29644-3 PMC9922304

[vbaf251-B11] Sharma A , Vans E, Shigemizu D et al Deepinsight: a methodology to transform a non-image data to an image for convolution neural network architecture. Sci Rep 2019;9:11399. 31388036 10.1038/s41598-019-47765-6 PMC6684600

[vbaf251-B12] Shen WX , Zeng X, Zhu F et al Out-of-the-box deep learning prediction of pharmaceutical properties by broadly learned knowledge-based molecular representations. Nat Mach Intell 2021;3:334–43.

[vbaf251-B13] Shin I , Kang K, Kim J et al AptaTrans: a deep neural network for predicting aptamer-protein interaction using pretrained encoders. BMC Bioinformatics 2023;24:447. 38012571 10.1186/s12859-023-05577-6 PMC10680337

[vbaf251-B14] Torrésani B. Time-Frequency and Time-Scale Analysis. Amsterdam, Netherlands: Academic Press, 1999.

[vbaf251-B15] Yang B , Zhang Z, Yang C-Q et al Identification of species by combining molecular and morphological data using convolutional neural networks. Syst Biol 2022;71:690–705. 34524452 10.1093/sysbio/syab076

[vbaf251-B16] Yoshimori A. Prediction of molecular properties using molecular topographic map. Molecules 2021;26:15. 10.3390/molecules26154475 PMC8348331 34361624

[vbaf251-B17] Zhang M , Zhou J, Wang X et al DeepBP: ensemble deep learning strategy for bioactive peptide prediction. BMC Bioinformatics 2024;25:352. 39528950 10.1186/s12859-024-05974-5 PMC11556071

[vbaf251-B18] Zhu Y , Brettin T, Xia F et al Converting tabular data into images for deep learning with convolutional neural networks. Sci Rep 2021;11:11325. 34059739 10.1038/s41598-021-90923-y PMC8166880

